# Adherence to the Mediterranean diet is positively associated with sperm motility: A cross-sectional analysis

**DOI:** 10.1038/s41598-019-39826-7

**Published:** 2019-03-04

**Authors:** Albert Salas-Huetos, Nancy Babio, Douglas T. Carrell, Mònica Bulló, Jordi Salas-Salvadó

**Affiliations:** 1 Universitat Rovira i Virgili, Biochemistry and Biotechnology Department, Human Nutrition Unit, Reus, Spain; 2 Institut d’Investigació Sanitària Pere i Virgili, Reus, Spain; 3 Consorcio CIBER, M.P., Fisiopatología de la Obesidad y Nutrición (CIBERobn), Instituto de Salud Carlos III (ISCIII), Madrid, Spain; 4 Division of Urology, Department of Surgery, University of Utah School of Medicine, Salt Lake City, UT USA; 5 Department of Human Genetics, University of Utah School of Medicine, Salt Lake City, UT USA; 6 Hospital Universitari Sant Joan de Reus, Reus, Spain

## Abstract

The aim of this cross-sectional analysis is to investigate the associations between the adherence to the Mediterranean Diet (MD) and semen quality parameters. To assess the adherence to the MD, the Trichopoulou score was used. Semen parameters were assessed as described in the 2010 WHO’s report and the results are showed across tertiles of MD adherence. A total of 106 participants were included. Compared to those in the lowest MD adherence tertile, participants in the top tertile had statistically significant higher BMI and waist circumference and consumed more energy, and also had statistically significant higher semen pH, and total sperm motility and progressive sperm motility percentages, and lower sperm immotility percentages. Moreover, percentage of total and progressive motility were significantly higher among those subjects in the higher adherence to MD in comparison with those in low-medium adherence category. The multivariable linear regression models evaluating the relationship between the sperm quality parameters and tertiles of MD adherence adjusted by age, energy and BMI showed that compared with the lowest tertile, men in the highest tertile had a higher percentage of total sperm motility [β non-standardized coefficient = 12.785]. These findings suggest that adherence to the MD was positively associated with sperm motility.

## Introduction

Male reproductive health is declining year by year raising serious concerns and implications about human fertility^1^. Nowadays infertility affects ∼15% of the world’s population and male factors are responsible for 40–50% of these cases^2,3^. Although it has been shown to be a problem worldwide, this decrease is stronger in certain geographic regions of the world, specifically in the developed and industrialized countries^4,5^. Therefore, enhancing research on causes of this decline is urgently needed. There are multiple possible causes of this decline, however, the most plausible seems to be related to environmental and lifestyle factors such as pollution^6^, smoking^7^, alcohol consumption^8^, lack of physical activity^9^, psychological stress^10^, overweight or obesity^11^, and unhealthy diets^12^.

Accumulating observational literature^12–15^ supports the hypothesis that specific foods can have beneficial (e.g. fish, shellfish and seafood, poultry, cereals, vegetables and fruits, and low-fat dairy) or deleterious (e.g. processed meat, soy foods, potatoes, full-fat dairy products, coffee, alcohol and sugar-sweetened beverages and sweets) effects on semen quality. Also, a recent meta-analysis of randomized clinical trials (RCTs)^16^ proposes that some nutrients and dietary supplements (e.g. omega-3, CoQ10, selenium, zinc and carnitines) could beneficially modulate sperm quality parameters and affect male fertility, even though, these last results must be cautiously interpreted due to the limited sample size of the meta-analyzed studies and the considerable observed inter-study heterogeneity. However, there are few studies focused on assessing the possible role of dietary patterns on semen quality parameters, especially in healthy and young population.

One of the healthiest dietary patterns, described as the Mediterranean diet (MD)^17,18,19^, is characterized by a high consumption of olive oil, fruit, nuts, legumes, vegetables and whole cereals; a moderate intake of fish, poultry and wine; and a low consumption of dairy products, red meat, processed meats, and sweets. Because this dietary pattern has been proved to have beneficial effects in several intermediate metabolic outcomes, such as inflammation, oxidative stress and insulin resistance, all of which are related to sperm function, we can hypothesize that adherence to the MD can have benefits in terms of semen quality parameters.

Therefore, the aim of the present study was to investigate the associations between the adherence to the MD pattern and several recognized semen quality parameters (e.g. semen volume and pH, total sperm count and concentration, sperm motility and vitality, and sperm morphology) in healthy and reproductive-age males, using a validated MD score.

## Results

A total of 244 subjects were assessed for eligibility, 119 were enrolled in the study and finally, 106 participants (89.1%) were included in the present analysis because a completed seminogram and dietary measures were obtained at baseline.

The mean age (±SD) of the study population (n = 106) was 24.7 (±4.7) years old. Most men had a normal BMI (n = 74, 69.8%), one participant was underweight (0.9%), 26 were overweight (24.5%), and 5 were obese (4.7%). The median and interquartile range (IQR) values for the semen parameters were: 73.8 × 10^6^ spz. (27.9 × 10^6^−125.5 × 10^6^) for total sperm count, 24.1 × 10^6^ spz./ml (10.8 × 10^6^−43.1 × 10^6^) for sperm concentration, 79.2% (72.3–84–8%) for sperm vitality, 66.3% (47.6–75.0%) form sperm total motility, 46.5% (28.7–57.8%) for sperm progressive motility and 6.4% (5.2–7.9%) for normal sperm morphology. Most men had at least one major semen analysis parameter (volume, total sperm count or concentration, vitality, motility or morphology) below the WHO 2010 reference values (58.5%).

The baseline characteristics of the participants according to the tertiles of MD are shown in Table 1. Compared to those in the lowest tertile, participants in the top tertile had statistically significant higher BMI and waist circumference and consumed more energy. Regarding the sperm parameters, the participants in the higher tertile, compared with the lowest tertile, had statistically significant higher pH (8 vs. 8.5, P-value = 0.023), higher total motility percentage (54.5% vs. 73.2%, P-value = 0.009), higher progressive motility percentage (40.8% vs. 52.2%, P-value = 0.013) and lower sperm immotility (34.3% vs. 29.0%, P-value = 0.045). No significant differences were shown in the rest of parameters.

**Table 1:** Baseline characteristics of the study population according to tertiles of Mediterranean diet adherence.

Variables	1^st^ Tertile n = 30 MD score ≤3	2^nd^ Tertile n = 49 MD score 4–5	3^rd^ Tertile n = 27 MD score ≥6	P-value
MD score*; median [Pc25-Pc75]	3 [2–3]^a^	4 [4–5]^b^	6 [6–7]^c^	<0.001
**Demographic parameters**				
Age (years); mean (±SD)	24.1 (±4.5)	24.1 (±4.6)	26.3 (±4.8)	0.100
Energy (kcal); mean (±SD)	2418 (±701)^a^	2860 (±836)^b,c^	3192 (±720)^c^	0.001
BMI (kg/m^2^); mean (±SD)	22.5 (±2.3)^a^	24.3 (±3.7)^b,c^	24.4 (±2.5)^c^	0.021
Waist circumference (cm); mean (±SD)	78.4 (±5.6)^a^	81.5 (±9.3)^a,b^	84.6 (±6.8)^b^	0.014
Systolic blood pressure (mmHg); mean (±SD)	126.3 (±11.4)	129.0 (±9.3)	127.0 (±11.0)	0.571
Diastolic blood pressure(mmHg); mean (±SD)	72.6 (±7.0)	72.1 (±9.1)	72.7 (±7.7)	0.946
**Blood parameters**				
Plasma glucose (mg/dl); mean (±SD)	88.0 (±9.3)	87.1 (±7.2)	88.5 (±9.1)	0.759
Total cholesterol (mg/dl); median [Pc25-Pc75]	170.8 [150.8–182.3]	170.8 [147.5–192.0]	169.0 [149.0–188.0]	0.878
HDL-c (mg/dl); median [Pc25-Pc75]	57.7 [54.8–66.3]	56.0 [47.5–66.0]	56.0 [48.0–59.0]	0.467
LDL-c (mg/dl); median [Pc25-Pc75]	96.7 [76.8–105.5]	96.7 [73.5–106.0]	93.0 [79.0–108.0]	0.911
VLDL-c (mg/dl); median [Pc25-Pc75]	14.1 [11.0–16.3]	15.0 [9.5–19.5]	13.0 [12.0–18.0]	0.827
Triglycerides (mg/dl); median [Pc25-Pc75]	75.0 [56.5–81.6]	76.0 [48.5–99.0]	66.0 [59.0–88.0]	0.739
Plasma insulin (mcUI/ml); mean (±SD)	6.1 (±3.7)	6.8 (±5.5)	6.2 (±5.7)	0.794
C-reactive protein (mg/dl); median [Pc25-Pc75]	0.2 [0.2–0.2]	0.2 [0.2–0.2]	0.2 [0.2–0.2]	0.985
Folate (ng/ml); median [Pc25-Pc75]	6.6 [5.7–8.9]	6.3 [4.7–6.9]	6.2 [4.6–8.1]	0.150
**Semen/sperm parameters**				
pH; median [Pc25-Pc75]	8.0 [8.0–8.5]^a^	8.0 [8.0–8.5]^a,b^	8.5 [8.0–8.5]^a,c^	0.023
Volume (ml); median [Pc25-Pc75]	2.8 [1.9–4.5]	3.0 [1.9–4.6]	3.3 [2.1–4.4]	0.852
Volume <1.5 ml; n (%)	3 (10)	8 (16.3)	4 (14.8)	0.731
Total sperm count (x10^6^); median [Pc25-Pc75]	76.6 [30.8–139.5]	65.0 [24.8–126.0]	79.0 [43.5–101.0]	0.675
Total sperm count <39 × 10^6^ spz; n (%)	9 (30)	19 (38.8)	6 (22.2)	0.321
Sperm concentration (x10^6^); median [Pc25-Pc75]	27.6 [9.1–53.7]	23.6 [10.0–38.1]	25.2 [16.1–35.6]	0.669
Sperm concentration <15 × 10^6^ spz/ml; n (%)	11 (36.7)	19 (38.8)	5 (18.5)	0.175
Vitality (%); median [Pc25-Pc75]	79.2 [69.4–82.2]	79.3 [72.7–84.2]	78.9 [72.7–87.9]	0.662
Vitality <58%; n (%)	3 (10)	2 (4.1)	3 (11.1)	0.451
Total motility (%); median [Pc25-Pc75]	54.5 [38.8–72.4]^a^	64.4 [43.8–72.7]^a,b^	73.2 [64.7–82.2]^c^	0.009
Total motility <40% motile; n (%)	8 (26.7)	7 (14.3)	2 (7.4)	0.127
Progressive motility (%); median [Pc25-Pc75]	40.8 [20.9–58.8]^a^	41.8 [25.9–53.5]^a,b^	52.2 [43.3–62.3]^c^	0.013
Progressive motility <32% motile; n (%)	10 (33.3)	17 (34.7)	4 (14.8)	0.160
Non-progressive motility (%); median [Pc25-Pc75]	11.1 [6.3–14.4]	12.1 [8.6–16.1]	12.1 [7.9–15.3]	0.674
Immotility (%); median [Pc25-Pc75]	34.3 [25.6–53.0]^a^	35.6 [27.3–56.2]^a,b^	29.0 [20.1–35.9]^a,c^	0.045
Morphology (%); median [Pc25-Pc75]	6.4 [4.4–8.0]	6.5 [5.6–7.9]	6.3 [5.0–8.0]	0.762
Morphology <4% normal; n (%)	5 (16.7)	5 (10.2)	2 (7.4)	0.515
Seminogram abnormality; n (%)	19 (63.3)	32 (65.3)	11 (40.7)	0.094

Abbreviations. BMI: Body-mass-index, HDL-c: High-density lipoprotein cholesterol, LDL-c: Low-density lipoprotein cholesterol, MD: Mediterranean diet, n: number of subjects, Pc: percentile, spz: spermatozoa, VLDL-c: very-low-density lipoprotein cholesterol, SD: standard deviation.

*MD score (Trichopoulou *et al*.)^18^.

Continuous variables were presented as means (±SD) or medians [25th-75th percentiles (Pc)] and categorical variables are presented as number (n) and percentages (%). Differences in variables across tertiles of MD adherence were tested using ANOVA test or Kruskal–Wallis test for continuous variables. Same superscripts in different columns denote non-significant differences, while different superscript letters denote statistically significant differences in a paired comparison (after multiple comparison post-hoc Bonferroni test). Pearson chi-squared test (Fisher’s exact test) was used to compare categorical variables.

Participants with higher adherence to MD score had a higher consumption of vegetables, legumes, fruits and nuts, cereals, fish and seafood. No significant differences across tertiles of MD adherence were detected in dairy products, meat, MUFA/SFA ratio or alcohol intake (Table 2).

**Table 2:** Group food consumption of the study population according to tertiles of Mediterranean diet adherence.

Variables	1^st^ Tertile n = 30 MD score ≤3	2^nd^ Tertile n = 49 MD score 4–5	3^rd^ Tertile n = 27 MD score ≥6	P-value
MD score*; median [Pc25-Pc75]	3 [2–3]^a^	4 [4–5]^b^	6 [6–7]^c^	<0.001
Vegetables (g/d); mean (±SD)	284.3 (±133.7)^a^	458.7 (±243.4)^b,c^	561.2 (±200.2)^c^	<0.001
Legumes (g/d); mean (±SD)	18.0 (±10.5)^a^	24.6 (±18.2)^a^	36.6 (±21.8)^b^	<0.001
Fruits and nuts (g/d); mean (±SD)	176.3 (±104.4)^a^	272.4 (±151.2)^b^	353.2 (±137.6)^c^	<0.001
Dairy products (g/d); mean (±SD)	361.8 (±225.6)	316.1 (±188.3)	331.7 (±308.1)	0.702
Cereals (g/d); mean (±SD)	119.2 (±74.4)^a^	162.3 (±86.3)^a,b^	177.7 (±99.3)^b^	0.029
Meat (g/d); mean (±SD)	194.9 (±68.7)	229.7 (±112.4)	212.4 (±104.0)	0.322
Fish and seafood (g/d); mean (±SD)	66.5 (±34.7)^a^	91.3 (±56.1)^a,b^	147.4 (±67.4)^c^	<0.001
MUFA/SFA (ratio/d); mean (±SD)	0.83 (±0.28)	0.82 (±0.23)	0.85 (±0.25)	0.911
Alcohol (g/d); mean (±SD)	7.29 (±12.15)	9.17 (±8.87)	10.93 (±6.78)	0.353

Abbreviations. g/d: grams per day, MD: Mediterranean diet, MUFA: monounsaturated fatty acids, Pc: percentile, SD: standard deviation, SFA: saturated fatty acids.

*MD score (Trichopoulou *et al*.)^18^.

Continuous variables were presented as means (±SD) or medians [25th-75th percentiles (Pc)]. Differences in variables across tertiles of MD adherence were tested using ANOVA test. Same superscripts in different columns denote non-significant differences while different superscript letters denote statistically significant differences in a paired comparison (after multiple comparison post-hoc Bonferroni test).

**Figure 1:**
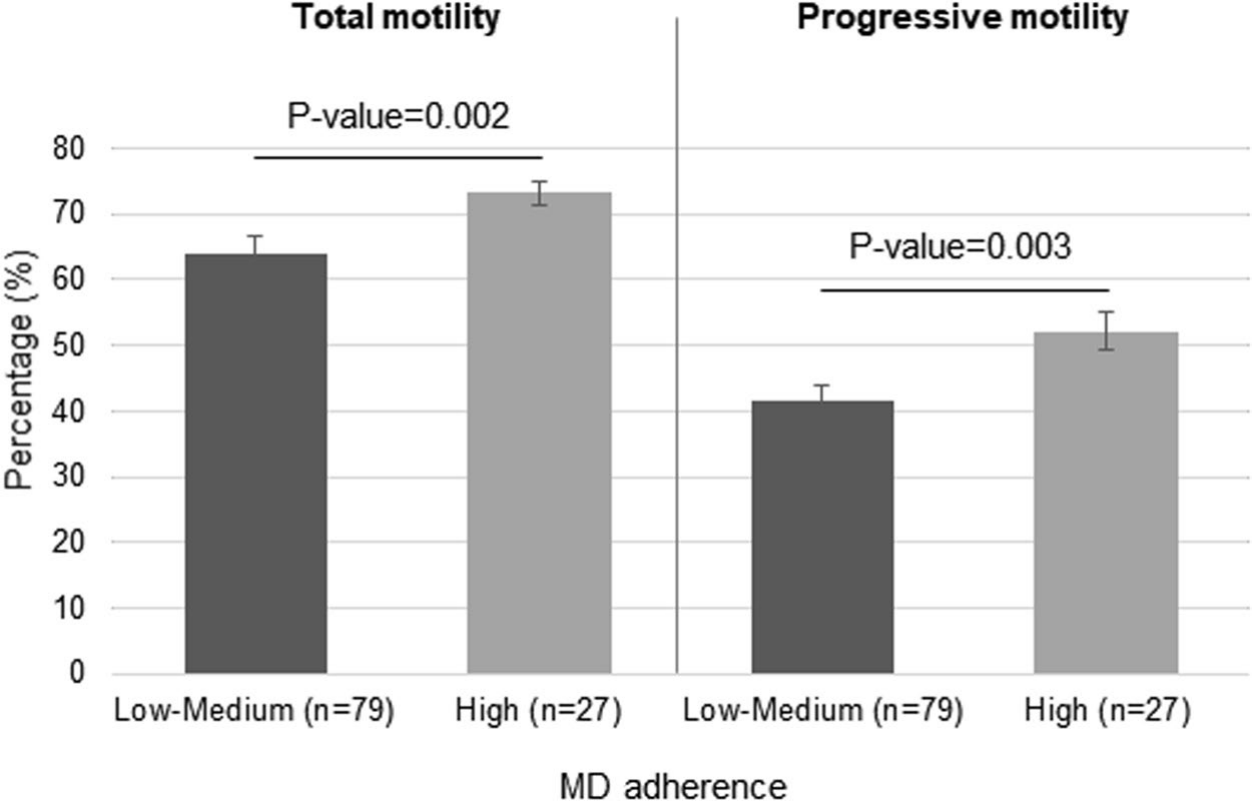
Median of total and progressive motility parameters according to Mediterranean diet adherence. Differences across the categorical MD adherence variable was tested using Mann-Whitney U test.

As shown in Fig. 1, in comparison with those participants that had a low-medium adherence to MD, the subjects with a high adherence had significantly higher percentages of total motility (P-value = 0.002) and progressive motility (P-value = 0.003).

Table 3 shows the multivariable linear regression models for evaluating the relationship between tertiles of MD adherence adjusted by age, energy and BMI as potential confounder factors, and several sperm parameters. A positive relationship was exhibited between MD adherence and the percentage of total sperm motility after adjusting for confounders, indicating an increase of a 12.8% of total sperm motility in those participants in the top tertile of MD adherence [β non-standardized coefficient = 12.785; P-value = 0.037].

**Table 3:** Multivariable linear regression models for tertiles of Mediterranean diet adherence and the principal sperm quality parameters.

	Total sperm count (x10^6^spz.)			Sperm concentration (x10^6^spz./ml)			Sperm vitality (%)		
	β non-standardized coefficient	Standard error	P-value	β non-standardized coefficient	Standard error	P-value	β non-standardized coefficient	Standard error	P-value
1^st^ Tertile	Reference			Reference			Reference		
2^nd^ Tertile	−20.573	18.927	0.280	−8.047	5.887	0.175	4.923	3.536	0.167
3^rd^ Tertile	−26.148	22.808	0.254	−8.132	7.094	0.254	6.669	4.261	0.121
Age (years)	2.179	1.655	0.191	0.310	0.515	0.549	−0.588	0.309	0.060
Energy (kcal)	0.005	0.010	0.584	−0.000	0.003	0.970	−0.002	0.002	0.195
BMI (kg/m^2^)	2.046	2.516	0.418	0.535	0.589	0.496	0.448	0.470	0.343
	R^2^x100 = 3.4%			R^2^x100 = 2.7%			R^2^x100 = 7.1%		
	F_5,105_ = 0.698			F_5,105_ = 0.545			F_5,105_ = 1.533		
	P-value = 0.629			P-value = 0.742			P-value = 0.186		
	**Total motility (%)**			**Progressive motility (%)**			**Normal sperm forms (%)**		
	β non-standardized coefficient	Standard error	P-value	β non-standardized coefficient	Standard error	P-value	β non-standardized coefficient	Standard error	P-value
1^st^ Tertile	Reference			Reference			Reference		
2^nd^ Tertile	1.803	5.010	0.720	−1.376	4.548	0.763	−0.162	0.632	0.799
3^rd^ Tertile	12.785	6.037	**0.037**	9.371	5.481	0.090	−0.345	0.762	0.652
Age (years)	0.224	0.438	0.610	0.435	0.398	0.277	−0.021	0.055	0.705
Energy (kcal)	0.000	0.003	0.941	0.001	0.002	0.764	0.001	0.000	0.068
BMI (kg/m^2^)	1.803	0.666	0.074	0.962	0.605	0.115	0.214	0.084	**0**.**012**
	R^2^x100 = 11.5%			R^2^x100 = 11.8%			R^2^x100 = 9.0%		
	F_5,105_ = 2.611			F_5,105_ = 2.678			F_5,105_ = 1.975		
	P-value = **0.029**			P-value = **0**.**026**			P-value = 0.089		

Abbreviations. BMI: Body-mass-index, spz: spermatozoa.

## Discussion

In this cross-sectional study conducted in healthy and young population we found that higher adherence to a Mediterranean diet, measured by the validated Trichopolou’s score, was associated with an increased sperm motility. Beyond the potential confounding factors considered in advance for the present study (all participants were non-smokers and healthy), this association was not only independent of the age and BMI of the participants, but also for the total energy consumption.

Our findings are consistent with previous epidemiologic observational studies^12^ that have reported a positive association between the consumption of individual components of a traditional Mediterranean diet such as fish and seafood^20^, poultry^21^, whole cereals^22^, vegetables^21^ and fruits^22^, low-fat dairy^23^, and the improvement of several sperm quality parameters. The results of the present study are also in line with other studies demonstrating that typical foods highly consumed in western dietary patterns, such us processed meat^24^, potatoes^25^, full-fat dairy and total dairy products^26^, alcohol^27^, sugar-sweetened beverages^28^ and sweets^21^, are detrimentally associated with some quality parameters of semen.

Our study has been based on the analysis of dietary pattern. Dietary pattern analysis has emerged as an alternative and complementary approach to examining the relationship between diet and the risk of health outcomes. Instead of looking at individual nutrients or foods, dietary pattern analysis examines the effects of overall diet. This approach takes into account possible food or nutrient interactions, and positive or negative synergistic effects^29^. Unfortunately, research assessing possible associations between dietary patterns and sperm quality outcomes is scarce and needs to be promoted in the future.

A recent systematic review and meta-analysis of six observational studies^30–35^ assessing the association between some *a posteriori* dietary patterns defined by factor analysis, and semen quality concluded that healthy eating patterns might have a positive association with sperm concentration, but not with other classical sperm quality parameters^14^. However, due to the small sample size and heterogeneity of the studies, the authors recommended caution in the interpretation of the results.

The traditional MD pattern has been considered one of the healthiest dietary patterns existing^36^. The MD has consistently been associated with broad healthy benefits on human health, especially in relation to protection against CVD and mortality^37^. Because this dietary pattern has also demonstrated beneficial effects on inflammation^38^, oxidation^39^, insulin sensitivity^40^, and endothelial function^41^ among others, we can hypothesize that it can have also benefits in terms of semen quality parameters and male fertility. However, whether adherence to the MD is associated with better semen profile remains largely unexplored.

To our knowledge, there was only one previously published study analyzing the association between MD adherence, defined *a priori*, and the quality of sperm^42^. In this cross-sectional study of 225 men from couples attending a fertility clinic in Athens, that has not been included in the aforementioned meta-analysis, men in the lowest tertile of the MD adherence score had higher likelihood of having abnormal sperm concentration, total sperm count and motility, compared to men in the highest tertile of the score. Therefore, our results confirm these previous reported results but only in relation to total and progressive sperm motility, an important parameter related to fertility.

In fact, healthy dietary patterns and specially the MD are rich in several nutrients that have been proven to have benefits in terms of sperm motility^43–48^ adding biological plausibility to the reported association. Only one study has demonstrated that adherence to a healthy dietary pattern is associated to sperm motility in *a posteriori* analysis. In the context of the Rochester Young Men’s cohort, adherence to a Prudent diet identified by factor analysis rich in fish, chicken, fruit, vegetables, legumes and whole grains was positively associated with progressive sperm motility^33^.

The principal strength of the present study is the originality of the work, because this is the first study exploring the association between the MD pattern and sperm quality parameters in a young and healthy population. The use of FFQs, as a comprehensive method to obtain detailed information about the habitual intake of foods allowed us to use a validated and widely used MD score measuring adherence to the MD^18^.

The main limitation of the study is the cross-sectional nature. Cross-sectional design could not determine whether MD adherence and sperm motility improvements are causally related. Future well-designed observational prospective studies and clinical trials on the current topic are therefore recommended. Although in our study we have excluded several potential confounding factors, these results should be interpreted with caution because of the multifactorial etiology of the sperm quality parameters. Moreover, we cannot discount other possible associations between the adherence to the MD and other sperm parameters by increasing the sample size or by repeating the semen analysis in order to decrease the biological variability. We cannot also discount some analytical error when measuring the sperm parameters, however we have minimized it through strong adherence to standardized protocols. Another limitation it that this study focuses on healthy men and therefore, the results cannot be extrapolated to other populations. Also, in some studies it has been shown that physical activity is associated with an improvement of sperm quality^49^. Unfortunately, we did not measure physical activity in our participants; therefore, we have not have controlled our models for this variable with the limitations that this entails in the interpretation of the findings. Finally, although an association between adherence to MD and sperm quality was detected, the highest probability of fecundability translation needs to be proven in the future.

In summary, in this healthy and young population, and using a cross-sectional analysis, we demonstrated that adherence to a MD was positively associated with sperm motility parameters. These findings suggest that compliance to a healthy diet may help to improve some semen quality parameters, especially those related to the sperm motility, one of the most important parameter related to fertility. Prospective studies and randomized clinical trials are warranted in the future to increase the scientific evidence in relation to the possible effect of diet on the sperm quality and fecundability.

## Material and Methods

### Study population

This cross-sectional analysis was conducted with baseline data of participants recruited between December 2015 and February 2017 in the FERTINUTS study, a parallel randomized clinical trial aimed to assess the effects of nut supplementation on sperm quality parameters. The design of the trial has been reported in detail elsewhere (ISRCTN12857940)^50^. Eligible participants were healthy and young men (aged 18–35 years old) reporting a western-style dietary pattern. Exclusion criteria included severe chronic illness, alcohol or drug abuse, frequent consumption or allergy of nuts, use of supplements (i.e. plant sterol, fish oil supplements, multivitamins, vitamin E or other antioxidant supplements), history of reproductive disorders or vasectomy, current smoking, or use of drugs for chronic diseases.

The protocol was approved by the Institutional Review Board of the Hospital Universitari Sant Joan de Reus in October 2015. The study was done according to the Declaration of Helsinki for Medical Research involving Human Subjects and all the participants provided a written informed consent.

### Dietary intake and adherence to the Mediterranean diet

Dietary intake was quantified by trained dietitians with a face-to-face 143-item, semi-quantitative, validated food frequency questionnaire (FFQ)^51^ over the past year and the mean of 3-day dietary records, including 2 workdays and a weekend day. In the FFQ, dietitians asked the participants about the frequency with which they consumed the different items: never, one to three times per month, once per week, two to four times per week, five to six times per week, once per day, two to three times per day, four to six times per day or more than six times per day. The responses were transformed to grams per day. Energy, nutrient intake, and food groups were estimated using Spanish food composition tables^52^.

In order to assess the adherence to the MD the Trichopoulou score was fitted from FFQ information^18^. Subjects were assigned a value of 0 if the consumption of components considered beneficial (vegetables, legumes, fruit and nuts, cereals, fish and seafood, monounsaturated -MUFA- to saturated fatty acids -SFA- ratio) was below the median, whereas individuals were assigned a value of 1 if they had a consumption of beneficial food at or above the median. Otherwise, people with consumption of components considered harmful (meat and dairy products) below the median were assigned a value of 1, whereas people with consumption at or above the median were assigned a value of 0. With regards to alcohol, a value of 1 was assigned to subjects consuming a moderate amount (i.e. between 10 and 30 g per day for men) and a value of 0 otherwise. Therefore, the total Trichopoulou score had a potential range from 0 (minimal adherence to MD) to 9 (maximal adherence to MD).

### General, anthropometric and blood measurements

General, anthropometrical variables and blood pressure were determined by trained staff at baseline. Briefly, body weight and height were measured using an electronic scale (TANITA TBF-300; Tanita), and BMI was calculated as weight in kilograms divided by squared height in meters. The commonly accepted BMI ranges are: underweight (<18.5 kg/m^2^), normal weight (18.5 to 25 kg/m^2^), overweight (25 to 30 kg/m^2^) and obese (>30 kg/m^2^). Waist circumference was measured to the nearest 0.5 cm midway between the lowest rib and the iliac crest with an anthropometric tape. Blood pressure was measured in duplicate with a 5 minutes interval by using a semiautomatic oscillometer (Omron HEM-705CP, Netherlands). Blood samples following 12 h fasting conditions were collected in order to measure the following parameters: plasma glucose, total serum cholesterol, high-density lipoprotein (HDL) cholesterol, low-density lipoprotein (LDL) cholesterol, very-low-density lipoprotein (VLDL) cholesterol, triglycerides, insulin, C-reactive protein, and folate (COBAS; Roche Diagnostics Ltd, UK).

### Sperm parameters analysis

Semen parameters were assessed as described in the 2010 World Health Organization’s report: “WHO laboratory manual for the examination and processing of human semen”^2^ and following the Björndahl checklist^53^ with at least 3 days of sexual abstinence. All analyses were done in fresh samples with a maximum of 60 minutes after collection. Briefly, semen volume and pH were measured after 30 minutes of liquefaction with a Pasteur pipette and pH-indicator strips, respectively. The lower reference limit for semen volume was 1.5 ml, and in the case of pH, 7.2 was used as a lower threshold value. Total sperm count, and concentration was determined with a 100-µm-deep haemocytometer chamber (Neubauer^®^), at ×400 magnification counting 200 spermatozoa. The lower reference limit for sperm concentration was 15 × 10^6^ spermatozoa per ml and for sperm count was 39 × 10^6^ spermatozoa per ejaculate. Sperm motility was assessed also at ×400 magnification (counting 200 spermatozoa) and permits to classify the spermatozoa as: a) Progressive motility (PR), b) Non-progressive motility (NP); and c) Immotility (IM). Total motility was expressed as PR + NP. The lower reference limit for total motility and progressive motility were 40% and 32%, respectively. Sperm vitality was estimated using eosin-nigrosine at ×1,000 magnification (evaluating 200 spermatozoa). The lower reference limit for vitality (membrane-intact spermatozoa) was 58%. Sperm morphology was assessed with Hemacolor^®^ (Millipore, Billerica, MA, USA) staining at ×1,000 magnification (assessing 200 spermatozoa), and expressed as percentage of normal forms. The lower reference limit for normal forms was 4%.

### Statistical analysis

The results are shown across tertiles of MD adherence. Continuous variables were presented as means and standard deviation (±SD) or medians [25th-75th percentiles (Pc)] based on the normal or non-normal distribution. Missing blood values for the patients who have all the seminogram and dietary information were imputed using the mean of the variables measured. Normal distribution and homogeneity of variances were evaluated using Kolmogorov-Smirnov and Levene’s test, respectively. Categorical variables were presented as number (n) and percentages (%). Differences between groups were assessed with ANOVA test when the variables were normally distributed and Kruskal-Wallis or Mann-Whitney U tests when they were not. A multiple comparison post-hoc Bonferroni test were used to perform pairwise comparisons between groups. The Pearson chi-squared test was used to compare categorical variables. Multivariate linear regression models (enter method) were fitted to assess the relationship between tertiles of MD adherence and the principal sperm quality parameters. Estimates with β coefficient and standard error were shown. The multivariate linear regression models included the following potential confounders: age (years), energy (kcal) and BMI (kg/m^2^). The goodness of fit was expressed as: R square multiply per 100 (R^2^x100) and the variance analysis of the model and its significance (F-value and P-value). All P-values are two-tailed at the <0.05 level. Statistical analyses were conducted with the IBM-SPSS statistical package (version 22.0, SPSS Inc., Chicago, IL, USA).

## Data Availability

The datasets generated during and/or analysed during the current study are available from the corresponding author on reasonable request.
